# Molecular Mechanisms of Acetaldehyde-Mediated Carcinogenesis in Squamous Epithelium

**DOI:** 10.3390/ijms18091943

**Published:** 2017-09-10

**Authors:** Ayaka Mizumoto, Shinya Ohashi, Kenshiro Hirohashi, Yusuke Amanuma, Tomonari Matsuda, Manabu Muto

**Affiliations:** 1 Department of Therapeutic Oncology, Graduate School of Medicine, Kyoto University, Kyoto 606-8507, Japan; ayaka@kuhp.kyoto-u.ac.jp (A.M.); ohashish@kuhp.kyoto-u.ac.jp (S.O.); yusuke12@kuhp.kyoto-u.ac.jp (Y.A.); 2 Department of Gastroenterology and Hepatology, Graduate School of Medicine, Kyoto University, Kyoto 606-8507, Japan; kenshiro@kuhp.kyoto-u.ac.jp; 3 Research Center for Environmental Quality Management, Kyoto University, Otsu 520-0811, Japan; matsuda.tomonari.8z@kyoto-u.ac.jp

**Keywords:** acetaldehyde, DNA adduct, esophageal squamous cell carcinoma, head and neck squamous cell carcinoma, DNA damage, cancer development, DNA repair pathway

## Abstract

Acetaldehyde is a highly reactive compound that causes various forms of damage to DNA, including DNA adducts, single- and/or double-strand breaks (DSBs), point mutations, sister chromatid exchanges (SCEs), and DNA–DNA cross-links. Among these, DNA adducts such as *N^2^*-ethylidene-2′-deoxyguanosine, *N^2^*-ethyl-2′-deoxyguanosine, *N^2^*-propano-2′-deoxyguanosine, and *N^2^*-etheno-2′-deoxyguanosine are central to acetaldehyde-mediated DNA damage because they are associated with the induction of DNA mutations, DNA–DNA cross-links, DSBs, and SCEs. Acetaldehyde is produced endogenously by alcohol metabolism and is catalyzed by aldehyde dehydrogenase 2 (ALDH2). Alcohol consumption increases blood and salivary acetaldehyde levels, especially in individuals with *ALDH2* polymorphisms, which are highly associated with the risk of squamous cell carcinomas in the upper aerodigestive tract. Based on extensive epidemiological evidence, the International Agency for Research on Cancer defined acetaldehyde associated with the consumption of alcoholic beverages as a “group 1 carcinogen” (definite carcinogen) for the esophagus and/or head and neck. In this article, we review recent advances from studies of acetaldehyde-mediated carcinogenesis in the squamous epithelium, focusing especially on acetaldehyde-mediated DNA adducts. We also give attention to research on acetaldehyde-mediated DNA repair pathways such as the Fanconi anemia pathway and refer to our studies on the prevention of acetaldehyde-mediated DNA damage.

## 1. Acetaldehyde, Acetaldehyde Metabolism, and Risk of Cancers

Acetaldehyde, a low molecular weight organic aldehyde with the formula CH_3_CHO, is a highly reactive compound that causes DNA damage [1,2]. It is found in food and drinks such as yogurt, ripe fruits, cheese, coffee, and alcoholic beverages [3,4], and in tobacco smoke [5]. In addition, acetaldehyde can be produced by microorganisms such as yeasts and bacteria in the human oral cavity [6,7,8]. Thus, acetaldehyde can be ingested orally in a variety of ways. In particular, alcoholic beverages such as Calvados and other spirits contain high quantities of “free” acetaldehyde (e.g., Calvados: 1781 ± 861 μM), and frequent consumption of these beverages is associated with an increased risk of esophageal squamous cell carcinoma (ESCC) [4,9], although “free” acetaldehyde present in alcoholic beverages appears to cause only a short time (1–2 min) direct exposure to the organs [10].

**Figure 1:**
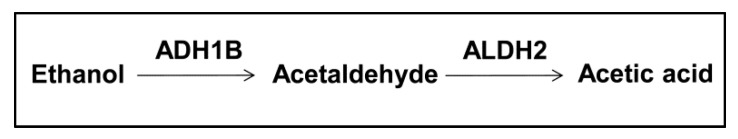
Ethanol and acetaldehyde metabolism after alcohol ingestion. Ethanol is metabolized to acetaldehyde by alcohol dehydrogenase 1B (ADH1B), and then acetaldehyde is degraded to acetic acid by aldehyde dehydrogenase 2 (ALDH2).

More importantly, acetaldehyde is also generated endogenously by alcohol metabolism. Ingested alcohol is absorbed from the upper gastrointestinal tract and transported to the liver, where it is mainly metabolized into acetaldehyde by alcohol dehydrogenase 1B (ADH1B), and then detoxified to acetic acid by aldehyde dehydrogenase 2 (ALDH2) (Figure 1) [11,12]. Genetic polymorphisms in *ADH1B* and/or *ALDH2* can result in different enzymatic activities that have a major impact on the risk of ESCC as well as head and neck squamous cell carcinoma (HNSCC) [13,14,15,16].

ADH1B has two alleles, ADH1B*1 (less active ADH1B) and ADH1B*2 (active ADH1B, Arg47His). Therefore, ADH1B is divided into three genotypes; ADH1B*1/*1, less active slow metabolizing ADH1B, and ADH1B*1/*2 and ADH1B*2/*2, active ADH1B [17]. Since alcohol metabolism is slow in individuals homozygous for ADH1B*1/*1, acetaldehyde remains in the body for a long time. Meta-analysis has shown that individuals with ADH1B*1/*1 have a 2.77- and 2.35-fold increased risk of ESCC [18] and HNSCC [19], respectively, compared with carriers of the ADH1B*2 allele (ADH1B*1/*2 and ADH1B*2/*2).

ALDH2 has two alleles, ALDH2*1 (active ALDH2) and ALDH2*2 (inactive ALDH2, Glu504Lys). As ALDH2 is a tetrameric enzyme and ALDH2*2 acts in a dominant negative manner, the phenotypic loss of ALDH2 activity is found in both heterozygous (ALDH2*1/*2) and homozygous (ALDH2*2/*2) genotypes [20,21]. Subsequently, ALDH2 genotypes are classified as follows: ALDH2*1/*1, active (100% activity) ALDH2; ALDH2*1/*2, inactive (< 10% activity) ALDH2; and ALDH2*2/*2, inactive (0% activity) ALDH2 [22]. Carriers of the ALDH2*2 allele (ALDH2*1/*2 and ALDH2*2/*2) account for approximately 40% of East Asian populations [23,24,25], whereas these genotypes are quite rare in Caucasoid or Negroid populations [26]. Meta-analysis has shown that individuals with ALDH2*1/*2 have a 7.12- and 1.83-fold increased risk of ESCC [14] and HNSCC [27], respectively, compared with carriers of ALDH2*1/*1. Moreover, alcoholics with the ALDH2*1/*2 genotype have a 13.5- and 18.52-fold increased risk of ESCC and HNSCC, respectively, compared with ALDH2*1/*1 genotypes [15].

Thus, extensive epidemiological evidence suggests that acetaldehyde is deeply involved in the carcinogenesis of the squamous epithelium of the esophagus, and head and neck. In addition, the International Agency for Research on Cancer has defined acetaldehyde associated with the consumption of alcoholic beverages as a “group 1 carcinogen” (definite carcinogen) for the esophagus and/or head and neck [28].

## 2. Field Cancerization in the Esophagus, and Head and Neck

**Figure 2:**
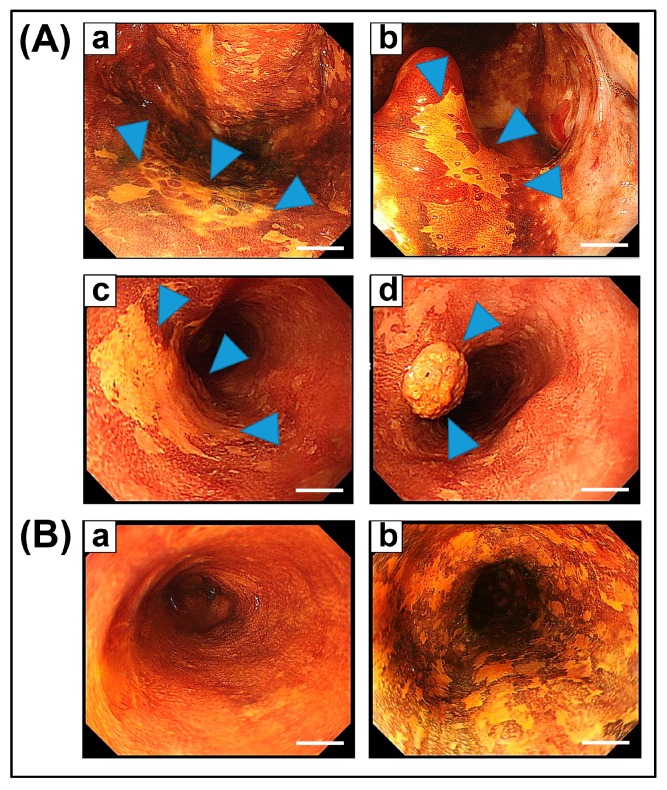
Lugol chromoendoscopic images. (**A**): “Field cancerization” in a patient with esophageal squamous cell carcinoma (ESCC) and head and neck squamous cell carcinoma (HNSCC) synchronously. Location of (**a**) oropharynx, (**b**) uvula, (**c**) upper thoracic esophagus, and (**d**) lower thoracic esophagus. Lesions are indicated by arrowheads; (**B**): (**a**) normal esophageal mucosa, (**b**) esophageal mucosa with multiple dysplastic lesions known as multiple Lugol-voiding lesions. Scale bar = 0.5 cm.

In some patients, ESCC occurs synchronously and/or metachronously in conjunction with HNSCC (Figure 2A) [12,29]. In such patients, widespread epithelial oncogenic alterations are frequently observed in the esophagus and can be visible as multiple Lugol-voiding lesions (LVLs) by Lugol chromoendoscopy (Figure 2B) [30,31]. Thus, multiple occurrences of neoplastic changes in the upper aerodigestive tract have been explained by the phenomenon of “field cancerization” [32]. We reported previously that the ALDH2*2 allele is the strongest contributing factor (OR: 17.6) for the development of multiple LVLs [29]. Our recent prospective cohort study also revealed that the severity of LVLs is associated with the amount of average alcohol consumption, and individuals with multiple LVLs in their esophagus are especially at high risk for metachronous multiple ESCC and HNSCC [33]. Thus, alcohol consumption in individuals with the ALDH2*2 allele is proven to be associated with the development of field cancerization in the esophagus, and head and neck.

## 3. Blood and Salivary Acetaldehyde Level after Alcohol Intake

Alcohol consumption increases acetaldehyde concentrations in the blood, saliva, and breath [29,34,35]. In particular, acetaldehyde concentration reaches a very high level in saliva compared with blood [6]. When ALDH2*1/*1 or ALDH2*1/*2 carriers drink 0.6 g ethanol/kg body weight, salivary acetaldehyde concentrations immediately reach 24 to 53 μM in ALDH2*1/*1 carriers and 37 to 76 μM in ALDH2*1/*2 carriers, respectively [36]. The reason for the high acetaldehyde concentrations in saliva is considered to be associated with the formation of acetaldehyde from ethanol via microbial [6] and/or mucosal ADH [37]. Moreover, secretion from salivary glands also influences acetaldehyde concentration in saliva. Indeed, alcohol drinking (0.5 g ethanol/kg body weight) increases acetaldehyde concentrations in parotid duct saliva on ALDH2*1/*2 carriers, while it does not affect those on ALDH2*1/*1 carriers [38]. Furthermore, breath acetaldehyde is also thought to dissolve into saliva. The acetaldehyde concentrations in the oral cavity thus produced are equivalent to the concentration that can induce DNA damage in vitro [6,38]. Therefore, alcohol consumption in ALDH2*1/*2 carriers could promote the direct contact of high acetaldehyde-containing saliva to the surface of the oropharynx, hypopharynx, and esophagus and has the potential to induce DNA damage in the squamous epithelium. Taken together, sustained high acetaldehyde-containing saliva is considered to play an important role in the carcinogenesis of upper digestive tract cancers and it could be involved in “field cancerization.”

## 4. Acetaldehyde Reacts with DNA to Form DNA Adducts

**Figure 3:**
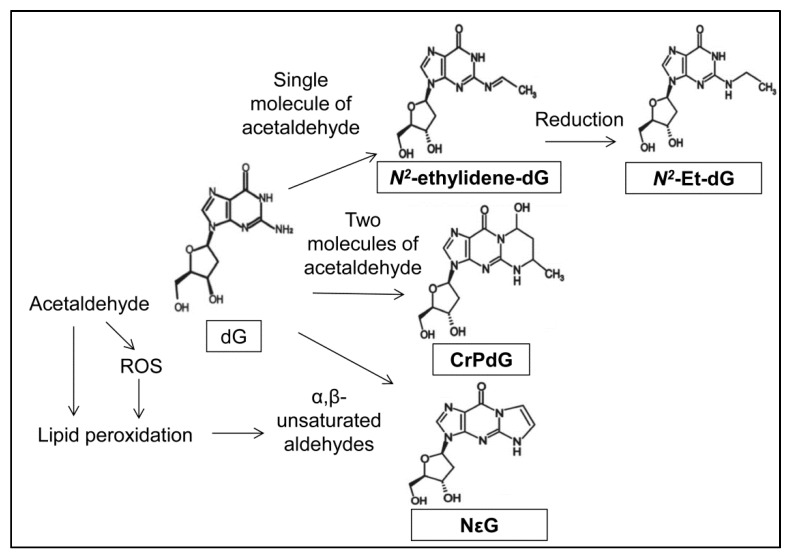
Formation of acetaldehyde-mediated DNA adducts. A single molecule of acetaldehyde reacts with deoxyguanosine (dG) to generate *N^2^*-ethylidene-2′-deoxyguanosine (*N^2^*-ethylidene-dG), which can be reduced to the stable adducts, *N^2^*-ethyl-2′-deoxyguanosine (*N^2^*-Et-dG). α-*S*- and α-*R*-methyl-γ-hydroxy-1, *N^2^*-propano-2′-deoxyguanosine (CrPdG) is derived from dG and two molecules of acetaldehyde. *N^2^*-etheno-2′-deoxyguanosine (NεG) is formed from dG and α,β-unsaturated aldehydes during lipid peroxidation, which is mediated by acetaldehyde or reactive oxygen species (ROS).

Acetaldehyde reacts directly with the exocyclic amino group of deoxyguanosine (dG) to form DNA adducts such as *N^2^*-ethylidene-2′-deoxyguanosine (*N^2^*-ethylidene-dG) [39], *N^2^*-ethyl-2′-deoxyguanosine (*N^2^*-Et-dG) [40,41], and α-*S*- and α-*R*-methyl-γ-hydroxy-1, *N^2^*-propano-2′-deoxyguanosine (CrPdG) (Figure 3) [39,42].

*N^2^*-ethylidene-dG is generated by a single molecule of acetaldehyde and is the most abundant DNA adduct derived from acetaldehyde [43]. *N^2^*-ethylidene-dG is unstable at the nucleoside level and is therefore difficult to measure [39]. *N^2^*-ethylidene-dG can be stabilized by the chemical reduction of the Schiff base to the stable product, *N^2^*-Et-dG. As endogenous *N^2^*-Et-dG is extremely low, the level of *N^2^*-Et-dG that is converted from *N^2^*-ethylidene-dG by chemical reduction (e.g., NaBH_3_CN) indicates the endogenous *N^2^*-ethylidene-dG level [44]. Thus, *N^2^*-ethylidene-dG is used for analysis of acetaldehyde-mediated DNA damage [43,45,46] as a biomarker for acetaldehyde-specific DNA damage [47]. Indeed, alcohol consumption increases oral *N^2^*-ethylidene-dG levels [48,49]. Furthermore, blood *N^2^*-ethylidene-dG levels are definitely increased by alcohol consumption [50] and/or tobacco smoking [51]. Additionally, blood *N^2^*-ethylidene-dG levels in alcoholics with the ALDH2*2 allele are higher than those with the ALDH2*1/*1 allele [46]. Importantly, alcohol consumption increases the esophageal *N^2^*-ethylidene-dG levels in *Aldh2*-knockout mice to a higher level than that of wild-type mice [47,52]. This evidence indicates that drinking alcohol definitely increases acetaldehyde exposure to the esophageal tissues in individuals with the ALDH2*2 allele.

CrPdG is generated by the reaction of two molecules of acetaldehyde with DNA [53] and exists in a ring-opened or ring-closed form [54,55]. Here, two molecules of acetaldehyde are converted into crotonaldehyde and then react with DNA to form CrPdG [56]. The levels of CrPdG are also related to the amount of acetaldehyde produced [57].

An ethenobase adduct, 1,*N^2^*-etheno-2′-deoxyguanosine (NεG), is generated in human cells treated with acetaldehyde [53]. NεG is a product from 2′-deoxyguanosine and α,β-unsaturated aldehydes that can be formed during lipid peroxidation mediated by acetaldehyde (Figure 3) [53,58]. As acetaldehyde induces reactive oxygen species (ROS) that leads to lipid peroxidation [59], generation of NεG can be triggered by acetaldehyde, ROS, or both.

## 5. DNA Adducts Induce Severe DNA Damage

*N^2^*-Et-dG blocks DNA synthesis and induces DNA mutations [60,61,62,63]. Moreover, *N^2^*-Et-dG inhibits translesion DNA synthesis (TLS), which leads to a majority of frameshift deletions and a minority of G:C > T:A transversions in human cells [62]. *N^2^*-Et-dG can rotate around the exocyclic nitrogen and the alpha carbon of acetaldehyde because it has a single bond, whereas *N^2^*-ethylidene-dG has a double bond, which makes it more hydrophobic than *N^2^*-Et-dG. These differences may result in significantly different mutagenic potential between *N^2^*-Et-dG and *N^2^*-ethylidene-dG [2].

CrPdG induces DNA interstrand [64] and intrastrand cross-links [65]. The ring-opened form of CrPdG can react with dG on the opposite strand of the DNA to form DNA interstrand cross-links [66]. A similar mechanism has been suggested for the formation of DNA intrastrand cross-links [2]. Whereas the ring-closed form of CrPdG would prevent Watson–Crick base pairing with cytosine in the anti conformation, Hoogsteen base pairing with cytosine would be possible in the *syn* conformation [55]. CrPdG-mediated disruption of the DNA replication process is thought to cause DNA damage [55,67,68,69].

NεG inhibits a replicative polymerase δ in complex with proliferating cell nuclear antigen (PCNA) while translesion polymerases η, ι, and κ can bypass the lesion with varying mutagenic consequences [70,71,72]. In cells, replication of a plasmid containing a site-specific NεG induces base-pair mutations at the NεG site as well as deletions, rearrangements, double mutants, and base-pair substitutions near the NεG site [73]. These mutations near the NεG site could be triggered by error-prone processing of DNA double-strand breaks (DSBs) resulting from a replication fork collapse caused by NεG [2]. Certainly, acetaldehyde blocks DNA replication and increases the level of phosphorylated histone H2AX (γ-H2AX), a DSB marker, in cells [74].

Acetaldehyde exposure of human cells increases rates of sister chromatid exchange (SCE) [75]. SCE is thought to result from replication-blocking DNA lesions [76]. Although CrPdGs, NεG, and interstrand cross-links are shown to inhibit replication, the adducts or cross-links that relate to the formation of SCEs have not been elucidated.

## 6. Carcinogenic Effects of Acetaldehyde

To elaborate on details mentioned previously in part, acetaldehyde causes DNA adducts [39,40,41,42], DNA single-strand breaks, DSBs [77], point mutations [69], SCEs [78,79,80], DNA–DNA cross-links [81], micronuclei [82], and gross chromosomal aberrations [65,80]. Accumulations of these genetic abnormalities are considered to proceed cancer development. Exposure of acetaldehyde directly induces mutations, most frequently G:C > A:T transitions in the TP53 gene [83]. This transition pattern is consistent with that found in a study of the HPRT reporter gene [69]. In addition, G:C > T:A transversions are the most frequent miscoding events induced by CrPdG, followed by G:C > C:G and G:C > A:T mutations [67,68,69]. This spectrum of mutations corresponds with the gene variation pattern observed in ESCC [84,85] and HNSCC [86]. Furthermore, inhalation of acetaldehyde causes nasal and respiratory squamous cell carcinoma in rats and hamsters [87,88]. These results indicate that acetaldehyde has direct carcinogenic effects in animals.

## 7. Repair Pathways of Acetaldehyde-Mediated DNA Damage

Recent research has revealed that cells coordinate multiple processes, such as the Fanconi anemia (FA) pathway, nucleotide excision repair (NER), homologous recombination (HR), TLS, base excision repair (BER), fork protection complex, and ATR-dependent cell cycle checkpoint activation, to prevent and repair acetaldehyde-mediated DNA damage [89].

The specific repair processes for *N^2^*-ethylidene-dG and *N^2^*-Et-dG remain unknown. The efforts to identify the repair mechanism for *N^2^*-Et-dG are reported to be unsuccessful [2,90].

The most plausible repair pathway of CrPdG is NER [91]. CrPdG generates interstrand cross-links [64], which can be repaired by the FA pathway [2]. This pathway is composed of at least 19 genes *(FANCA*, *B*, *C*, *D1*, *D2*, *E–G*, *I*, *J*, *L–T*) and the deficiency of these genes can cause FA [92]. FANCA, B, C, E–G, L, and M form a core complex at the site of interstrand cross-links and then promote ubiquitination of the FANCD2–FANCI complex. This ubiquitination leads to the activation of downstream effector proteins, FANCD1, O, P, and Q. They promote the nucleolytic processing of interstrand cross-links, followed by DNA repair via HR [93,94,95,96,97]. Indeed, the FA–BRCA network is activated when cells are treated with ethanol or aldehyde [98,99]. Cells derived from an FA patient are hypersensitive to acetaldehyde exposure [99,100]. Cells deficient for FANCG, FANCQ, or HR protein Rad51D also show many chromosomal aberrations in response to acetaldehyde, while cells deficient for BER and nonhomologous end-joining show subtle increases in chromosome aberration [101,102]. In vivo, when mice with disrupted *Aldh2* locus (*Aldh2*^+/−^ or *Aldh2*^−/−^) and *Fancd2* heterozygosity (*Fancd2*^+/−^) are crossed and then challenged with ethanol exposure, the numbers of double-knockout offspring (*Aldh2*^−/−^, *Fancd2*^−/−^) are significantly reduced [103]. Treatment with ethanol in adult double-knockout mice (*Aldh2*^−/−^, *Fancd2*^−/−^) results in dramatic reductions of bone marrow cells. Moreover, these mice develop leukemia, even without ethanol administration [103]. These results indicate that Fancd2 plays an important role in the protection from acetaldehyde-induced genotoxicity.

Acetaldehyde-mediated DSB is repaired by HR [74]. Acetaldehyde accumulates γ-H2AX, which colocalizes with foci of the HR protein Rad51 in cells [74]. Moreover, recombination-defective cells are hypersensitive to acetaldehyde [74].

## 8. Prevention of Acetaldehyde-Mediated DNA Damage

Acetaldehyde-mediated DNA damage is influenced by ALDH2 expression level [52]. ALDH2 is known to express in various tissues including the liver, kidney, muscle, and heart [104]. Recently, we found that alcohol consumption in mice promoted ALDH2 protein production in esophageal epithelium [52]. In vitro experiments revealed that ALDH2 is induced by acetaldehyde exposure in esophageal keratinocytes. ALDH2 knockdown resulted in an increase of susceptibility to acetaldehyde. Conversely, ALDH2 overexpression prevented acetaldehyde-mediated DNA damage in esophageal keratinocytes, although overexpression of mutant ALDH2 (ALDH2*2) offered no protection. Thus, enhancement of ALDH2 expression level may prevent acetaldehyde-mediated DNA damage.

## 9. Conclusions

**Figure 4:**
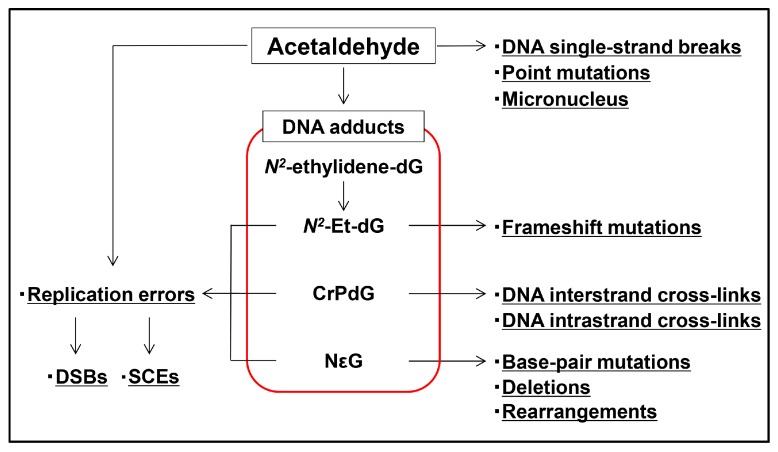
Summary of acetaldehyde-mediated DNA damage. Acetaldehyde causes DNA adducts, DNA single-strand breaks, DNA double-strand breaks (DSBs), point mutations, micronuclei, frameshift mutations, base-pair mutations, deletions, DNA–DNA interstrand or intrastrand cross-links, rearrangements, and sister chromatid exchanges (SCEs). DNA adducts are considered to be partly (but deeply) involved in their formation.

Previous studies have provided substantial evidence that acetaldehyde induces various forms of DNA damage leading to cancer development (Figure 4). DNA adduct formation might be the key to acetaldehyde-mediated DNA damage; however, the role of DNA adducts in carcinogenesis has not been completely elucidated. Further studies are necessary to reveal the complete mechanisms of acetaldehyde-mediated cancer development.

## Abbreviations

ADH1B	Alcohol dehydrogenase 1B
ALDH2	Aldehyde dehydrogenase 2
BER	Base excision repair
CrPdG	α-*S*- and α-*R*-methyl-γ-hydroxy-1, *N^2^*-propano-2′-deoxyguanosine
dG	Deoxyguanosine
DSB	Double-strand break
ESCC	Esophageal squamous cell carcinoma
FA	Fanconi anemia
HNSCC	Head and neck squamous cell carcinoma
HR	Homologous recombination
LVL	Lugol-voiding lesion
NER	Nucleotide excision repair
*N^2^*-Et-dG	*N^2^*-ethyl-2′-deoxyguanosine
*N^2^*-ethylidene-dG	*N^2^*-ethylidene-2′-deoxyguanosine
NεG	1,*N^2^*-etheno-2′-deoxyguanosine
PCNA	Proliferating cell nuclear antigen
ROS	Reactive oxygen species
SCE	Sister chromatid exchange
TLS	Translesion DNA synthesis
γ-H2AX	Phosphorylated histone H2AX
